# A marine zooplankton community vertically structured by light across diel to interannual timescales

**DOI:** 10.1098/rsbl.2020.0810

**Published:** 2021-02-24

**Authors:** Laura Hobbs, Neil S. Banas, Jonathan H. Cohen, Finlo R. Cottier, Jørgen Berge, Øystein Varpe

**Affiliations:** ^1^ Department of Mathematics and Statistics, University of Strathclyde, Glasgow G1 1XH, UK; ^2^ Scottish Association for Marine Science, Oban, Argyll PA37 1QA, UK; ^3^ School of Marine Science and Policy, University of Delaware, 700 Pilottown Road, Lewes, DE, USA; ^4^ Faculty for Biosciences, Fisheries and Economics, Department for Arctic and Marine Biology, UiT, The Arctic University of Norway, 9037 Tromsø, Norway; ^5^ Department of Arctic Biology, University Centre in Svalbard, Pb 156, N-9171 Longyearbyen, Norway; ^6^ Department of Biology and Technology, Centre of Autonomous Marine Operations and Systems, Norwegian University of Science and Technology, N-7491 Trondheim, Norway; ^7^ Department of Biological Sciences, University of Bergen, 5020 Bergen, Norway; ^8^ Norwegian Institute for Nature Research, 5006 Bergen, Norway

**Keywords:** Arctic, zooplankton, isolume, predation, migration

## Abstract

The predation risk of many aquatic taxa is dominated by visually searching predators, commonly a function of ambient light. Several studies propose that changes in visual predation will become a major climate-change impact on polar marine ecosystems. The High Arctic experiences extreme seasonality in the light environment, from 24 h light to 24 h darkness, and therefore provides a natural laboratory for studying light and predation risk over diel to seasonal timescales. Here, we show that zooplankton (observed using acoustics) in an Arctic fjord position themselves vertically in relation to light. A single isolume (depth-varying line of constant light intensity, the value of which is set at the lower limit of photobehaviour reponses of *Calanus* spp. and krill) forms a ceiling on zooplankton distribution. The vertical distribution is structured by light across timescales, from the deepening of zooplankton populations at midday as the sun rises in spring, to the depth to which zooplankton ascend to feed during diel vertical migration. These results suggest that zooplankton might already follow a foraging strategy that will keep visual predation risk roughly constant under changing light conditions, such as those caused by the reduction of sea ice, but likely with energetic costs such as lost feeding opportunities as a result of altered habitat use.

## 1. Introduction

Light influences zooplankton ecology in myriad ways, including prey availability, by limiting the initiation of the spring phytoplankton bloom, and mortality through visual predation. Zooplankton are, predominantly, negatively phototactic [1], migrating to depth during daylight to avoid the threat of visual predation and surfacing at night to feed (diel vertical migration, DVM [2]). Consequently, fitness through the water column is primarily governed by light through the balance of predation risk and prey availability [3–5]. The vertical distributions of zooplankton have consequences for predator–prey interactions [6], vertical carbon export [7] and energy transfer, and are important to quantify for implementation in behavioural, ecological and biological models [8–11].

The Arctic Ocean has a highly seasonal light environment (24 h of daylight in summer, and 24 h of ‘darkness’ in winter), and therefore presents a natural laboratory for observations of population responses to light. The migrations of zooplankton are seen to mirror these changes in the lightscape, sampled mostly using nets and active acoustic approaches [12], but also cameras [13]. During spring and autumn, there is a strong day–night light cycle, resulting in synchronized DVM as seen at mid-latitudes [12]. In summer, with no safe time to surface (in terms of visual predation), zooplankton make random foraging trips to the surface [14] rather than migrating as a population [12]. In winter, the underwater light climate, the ‘lightscape’, is controlled primarily by low-level sunlight and moonlight, and secondarily by aurora and bioluminescence [15]. Many zooplankton (such as *Calanus* spp.) often enter diapause at this time of year [16]. Full-depth synchronized DVM stops for a period of time at higher latitudes [17], and population-based zooplankton migrations become synchronized with lunar cycles [18–20], or are solar-driven at shallow depths [21].

Here, we use acoustic data (with the expected signal to be dominated by *Calanus* spp., krill (*Meganyctiphanes norvegica*, *Thyanoessa* spp.) and *Themisto* spp. [22]) from 3 years in an ice-free High-Arctic fjord to define the vertical positioning of a zooplankton community. We quantify the lightscape using downwelling solar irradiance and chlorophyll-*a* concentration (Chl-a), and describe the ways in which light mediates the vertical distribution of zooplankton in the context of predation risk. We select a light level that we expect to be meaningful in terms of light sensitivity (the lower limit of photobehaviour for the target species in the acoustics), and describe the vertical distribution of zooplankton in response to how the depth of this light level varies on diel, seasonal and interannual timescales.

## 2. Material and methods

**Figure 1.**
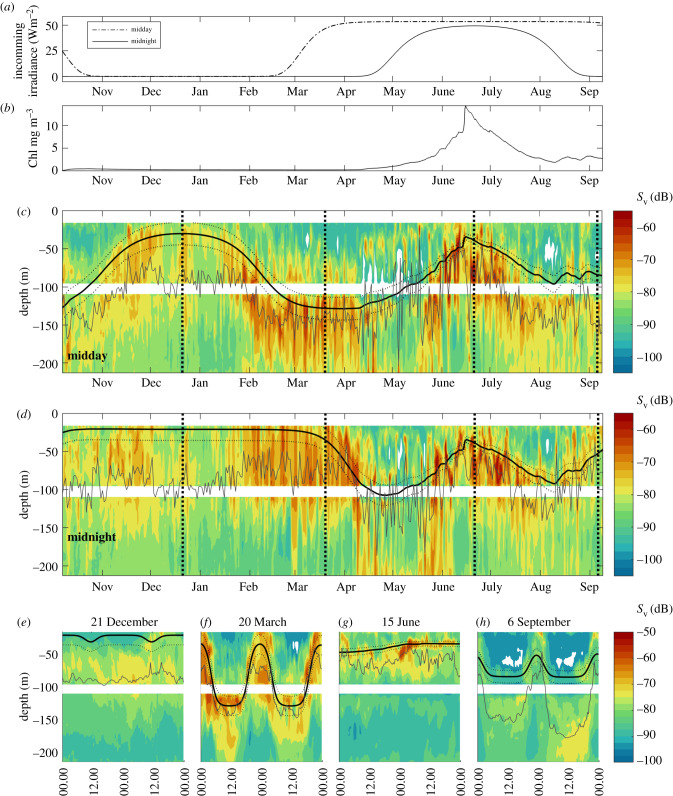
The seasonal and diel response of the meso-zooplankton community (observed using ADCP data (mean volume backscattering strength, *S*_v_ (dB))) to ambient light. All data acquired from 2013 to 2014. (*a*) Modelled incoming irradiance at midday (dot–dashed) and midnight (solid) at the sea surface; (*b*) chlorophyll-*a* concentration, as measured by a fluorometer at 38 m; (*c*–*h*) acoustic backscatter measured using two ADCPs at approximately 100 m. Dotted black lines show the depth of isolumes within the range of copepod and krill lower limit photobehaviour thresholds (10^−8^–10^−6^ µmol photons m^−2^ s^−1^ [26,27]), while the solid black line is the midpoint of this range (10^−7^ µmol photons m^−2^ s^−1^). (*c*,*d*) The full seasonal cycle, with backscatter data at local midday (*c*) and midnight (*d*). (*e*–*h*) Diel behaviour, with depth extracted for 48 h periods centred on (*e*) 21 December (winter solstice); (*f*) 20 March (spring equinox); (*g*) 15 June (near summer solstice, peak of spring bloom); (*h*) 6 September (closest data available to the autumn equinox). Grey lines on (*c*–*h*) indicate the depth of the centre of mass of backscatter. Vertical dashed lines on (*c*,*d*) correspond to dates of data extraction for (*e*–*h*).

We use 3 years of data from an oceanographic mooring in Kongsfjorden, Svalbard (78°N 11°E) for approximately 12 months in 2007–2008, 2008–2009 and 2013–2014. On each mooring, an upward-looking 300 kHz RDI acoustic Doppler current profiler (ADCP) was installed at approximately 100 m (bottom depth at the mooring was approx. 230 m). In 2013–2014, an additional downward-looking ADCP was deployed at a similar depth. ADCP data were processed to acoustic volume backscattering strength (*S*_v_, dB) [23], an estimate of the biomass of zooplankton present [24]. We calculate the centre of mass of acoustic backscatter using methods in [25], and use cumulative distribution to quantify the amount of zooplankton remaining below the isolume. We estimated light at the surface using a simplified model (figure 1*a*), and modelled underwater light as a function of depth using Chl-a concentration (figure 1*b*) as a shading component [28]. We selected an isolume (depth of continuous light intensity) of 10^−7^ µmol photons m^−2^ s^−1^, a midpoint of ranges published for the lower limit of photobehaviour for the likely target species in the backscatter signal [22], copepods (10^−8^ to 10^−6^ μmol photons m^−2^ s^−1^ [26]) and krill (10^−7^ to 10^−6^ µmol photons m^−2^ s^−1^ [27]). We use Lomb–Scargle periodograms [19] to test periodicity (as a proxy for synchronized DVM) during different light regimes across the year. More details on methodology are available in the electronic supplementary material.

## 3. Results

**Figure 2.**
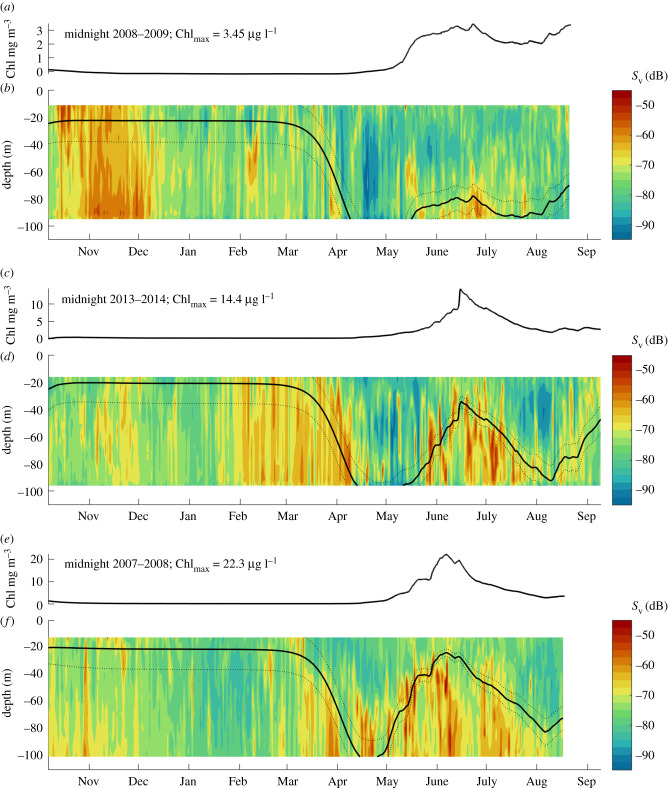
Interannual variation in the seasonal response to light. (*a*,*c*,*e*) Chlorophyll time series; (*b*,*d*,*f*) ADCP data (mean volume backscattering strength, *S*_v_ (dB)) at midnight from the top approximately 100 m, with isolumes as per figure 1. Data are shown from 3 years in Kongsfjorden. Chl_max_ indicates the maximum Chl-a concentration each year as a way of comparing spring bloom (and therefore shading) magnitude, with plots ordered by Chl-a magnitude.

The position of zooplankton is vertically closely related to isolumes (figures 1 and 2). We demonstrate the full-depth response using a single year of data (2013–2014, figure 1), and interannual variation in the top approximately 100 m (figure 2). There are seasonal (figure 1*a–d*), diel (figure 1*e*–*h*) and interannual (figure 2) responses of zooplankton vertical positioning to light, and we find that the shallow limit of the scattering layer is well-described by the 10^−7^ isolume in all cases. The 75th percentile of backscatter intensity (indicative of zooplankton biomass [24]) sits below the 10^−7^ isolume in 70 and 88% of observations at night and day, respectively (rising to 73 and 92% when we exclude the period in spring (26 April to 20 May) when the scattering layer is poorly defined).

In November to January there is a low abundance of zooplankton, but those present sit in the top 100 m, below the isolume both at midnight and at midday (figure 1*c*,*d*). There are small diel variations (approx. 10 m) in the depth of the isolume owing to the background solar cycle even at the winter solstice (figure 1*e*), although these short-term changes are not reflected in the backscatter. The depth of the 10^−7^ isolume in winter remains consistent (21–23 m) across years (figure 2), owing to consistent solar cycles and no phytoplankton shading. In January–March, the rising sun causes a deepening of the daytime isolume, which is tracked by the scattering layer (figure 1*c*). In spring, DVM (both night-time feeding and daytime refuge depths) tracks the isolume (figure 1*c*,*d,f*). There is low backscatter throughout the water column in May (figure 1*c*,*d*). The isolume shallows from May onwards (figures 1*c*,*d* and 2) as a result of shading by Chl-a [28] and this is reflected in the backscatter, which tracks the isolume towards the surface at midday and midnight (figure 1*c*,*d*,*g*). No synchronized DVM is observed in mid-summer (figure 1*g*). As the Chl-a concentration reduces in July (figure 1*b*), the isolume deepens, again reflected in the scattering layer depth (figure 1*c*,*d*). In September, the isolume shallows slightly as a result of a lower magnitude autumn phytoplankton bloom (figures 1*c*,*d* and 2) and reducing incoming irradiance (figure 1*a*). DVM is observed around the autumn equinox (figure 1*h*), but the overall diel variation in backscatter is much less than observed in spring (figure 1*f*). All interpretations of synchronized DVM are further evidenced through periodicity analysis in electronic supplementary material, figure S2.

The varying depth of the 10^−7^ isolume, caused by chlorophyll bloom magnitude and timing, also explains inter-annual differences in the vertical distribution of zooplankton (figure 2). In 2013–2014 and 2007–2008, high chlorophyll magnitude results in a shallower 10^−7^ isolume during the spring bloom. In 2008–2009, the magnitude of the spring bloom is much lower, deepening the isolume, and the scattering signal in the top 80 m is extremely low.

## 4. Discussion

Here, we have presented robust evidence for the role of light in determining the vertical positioning of zooplankton in the Arctic. A common isolume, selected here to represent the lower limit of photobehaviour threshold for Arctic zooplankton targeted by the ADCP (*Calanus* spp. and krill (*M. norvegica*)), sets a soft upper limit on zooplankton distribution on diel, seasonal and interannual timescales. The scattering layer (determined visually and by using the centre of mass) can be found at any depth below this limit (such as in diapause), but the isolume represents a boundary under which most of the zooplankton remain. The same isolume explains the depth of DVM in spring and autumn (figure 1*f*,*h*), and the lack of synchronized DVM in winter and summer (figure 1*e*,*g*). At these times of small or absent diel variation in incident light and overall low incoming light intensities (a consequence of low solar altitude in the winter (figure 1*a*), and shading from phytoplankton in the summer (figure 1*b*)), zooplankton instead occupy the upper 100 m. During the winter, we expect that zooplankton are making small (less than 10 m) migrations in the surface [21] but these are not detectable using ADCPs owing to acoustic interference at the air–sea interface.

The Arctic lightscape is changing [29], and we expect isolumes to deepen with sea ice decline. Although the results presented here are from an ice-free location, sea ice is known to have an impact on vertical migration through the modification of the light climate [12,17]. A lightening of the Arctic might increase the predation efficiency of planktivorous fish [30]. However, if the dominant polar zooplankton have evolved to avoid the layers above a certain visual sensitivity, then this negative-phototactic behaviour might buffer the heightened predation risk in the future Arctic, but at the cost of lost foraging opportunities as zooplankton are ‘pushed out’ of foraging grounds by prioritizing lowering predation mortality over energy intake. Disruption of habitat use in this way has been observed at other trophic levels [31], and habitat constriction is also seen through changes in oxygen levels [32]. Evidence of deep zooplankton distribution is seen in May–July of 2009 (figure 2*b*), when the top 80 m is completely devoid of zooplankton even though this is the time of maximum prey availability (figure 2*a*) with a shallow Chl-a max [33] . Note that in regions where zooplankton are not able to access dark enough depth layers, owing to a bathymetric constraint, there is no way to avoid increased visual predation through vertical migration (topographic trapping [34]), and thus future change in trophic coupling via these mechanisms may vary between shallow and deep Arctic habitats. Note also that visual sensitivity changes with temperature and oxygen levels [35] adding further complexity to estimates of future change.

As discussed above, optimal foraging strategy is usually theorized as a balance between risk and reward, expected mortality and expected energy gain. It is difficult to determine from acoustic or other abundance-versus-depth observations alone whether the vertical movement in the zooplankton is driven by the ‘risk’ or the ‘reward’ side of this balance. For example, we found that zooplankton vertical distribution varies in conjunction with observed interannual variation in bloom magnitude and timing, with the scattering layer being deeper in years of low bloom magnitude. One could hypothesize that this is driven by variation in ‘reward’: low prey abundance is less worth taking risks for, even if the risk is constant. However, we suggest that the interannual variation in behaviour can be explained more parsimoniously as a response to a deepening isolume: a single negative-phototactic behavioural rule can explain both the avoidance of the surface layer during the weak summer 2008–2009 bloom, and the active occupation of the surface layer during the even worse prey conditions of winter 2008–2009 and 2013–2014 (figure 2*a*–*d*). Summer phytoplankton blooms affect zooplankton fitness both by fuelling growth and by shading the water column and reducing light and risk, and assessing the relative importance of these effects in a changing Arctic will require more detailed energetic and physiological studies, both observational and model-based.

Here, we applied single frequency acoustics to observe a mixed zooplankton community, composed of several taxa. We anticipate that each of these taxa will have specific isolumes to which they respond, a consequence of visual physiology and perceived predation threat [36] as a function of body size or other individual states [37]. We note that the 10^−7^ isolume does not perfectly limit the vertical distribution of backscatter, only the majority of it. In all seasons and years, there is evidence of zooplankton above the isolume, although this appears temporally patchy. The majority of zooplankton (measured using the 75th percentile of cumulative backscatter distribution) sit below the 10^−7^ isolume, except during the low backscatter period around May. We suggest two hypotheses for the observations of zooplankton not remaining below the isolume: (i) zooplankters exhibit state-dependent behaviours, changing with individual variability, such as visual sensitivity, size or lipid reserves, or (ii) the isolume is not perfectly quantified here owing to other shading components such as cloud, run-off etc. or alternative sources of light such as the moon and aurora in the winter (we only considered sunlight owing to our focus on annual behaviours). Furthermore, we calibrated the Chl-a concentration time series using single-point measurements taken in the approximate location of the mooring, and therefore not an exact representation of the *in situ* Chl-a conditions. However, we find that the uncertainty in isolume depth that would follow from adjusting the estimated Chl-a concentration by ±50% is much smaller (approx. 30–40 m, electronic supplementary material, figure S3) than the observed change in isolume depth on a seasonal and interannual timescale (approx. 100 m, figures 1 and 2). With both hypotheses, we recommend further studies to identify individual variability in vertical positioning, and better quantification of the underwater light environment. We suggest further laboratory experiments to determine diel and seasonal variation in visual sensitivity, and the application of technology such as multi-frequency acoustics to determine the depth distribution of different taxonomic groups.

These results provide clear evidence for zooplankton communities following isolumes [3] across diel, as also observed in Greenland [13], and seasonal timescales in the Arctic. Our data suggest that predation risk will not necessarily change with a more illuminated Arctic, but there will be other consequences for zooplankton populations such as being pushed out of foraging depths and reducing food intake. This reduction in population growth has the potential to be balanced by increasing phytoplankton biomass [38], which will increase energy intake, and also reduce the effect of sea ice decline on isolume depth through the shading effect.

This study demonstrates a consistent and ecologically significant response of zooplankton to ambient light across many years. The methods developed here should be used to examine the spatial variation that exists on oceanic scales, and ideally be combined with methods able to capture the state of individual zooplankters (e.g. body condition). If the response of scattering layers to isolumes is found to hold on a pan-Arctic scale, it will provide a powerful predictive tool for understanding the consequences of sea ice loss and changes in primary production for the vertical distribution, and ultimately predation risk and foraging efficiency, of pelagic ecosystems.

## Data accessibility

All data and scripts used for plotting are available from the Dryad Repository (doi:10.5061/dryad.zw3r22878) [39]. We include .mat files for data, and .m scripts for analysis and plotting. In addition to this, all data are available as .csv files.
